# Drop and Swell: Unanticipated Corneal Edema From Netarsudil Therapy

**DOI:** 10.7759/cureus.73376

**Published:** 2024-11-10

**Authors:** Gufran A Kamdar, Surbhi A Chodvadiya, Radhika Paranjpe

**Affiliations:** 1 Ophthalmology, Dr. D. Y. Patil Medical College, Hospital and Research Centre, Pune, IND

**Keywords:** asoct (anterior segment optical coherence tomography), central retinal artery occlusion (crao), intraocular pressure (iop), neovascular glaucoma (nvg), slit lamp examination

## Abstract

Neovascular glaucoma (NVG) poses significant treatment challenges, often necessitating complex medication regimens to manage intraocular pressure (IOP). This report details a 65-year-old male with NVG secondary to central retinal artery occlusion (CRAO), who developed corneal epithelial bullae as a rare side effect of topical netarsudil therapy. Despite this complication, a continued treatment led to the gradual resolution of corneal lesions while maintaining controlled IOP. This case underscores the importance of vigilant monitoring for adverse effects and highlights the need for further research into netarsudil's safety profile in NVG management.

## Introduction

Netarsudil, a novel Rho-associated protein kinase (ROCK) inhibitor, has emerged as a promising therapeutic agent for lowering intraocular pressure (IOP) in patients with open-angle glaucoma and ocular hypertension. Its dual mechanism of action, involving enhancement of trabecular meshwork outflow and reduction of aqueous humor production, addresses unmet needs in the management of elevated IOP. Despite its efficacy, netarsudil is not without adverse effects, commonly manifesting as conjunctival hyperemia, subconjunctival hemorrhage, and corneal verticillata [1-3]. However, this case report highlights a rare and unexpected complication associated with netarsudil therapy bullous epithelial edema of the cornea [4].

## Case presentation

A 65-year-old male with a 20-year history of diabetes mellitus and irregular glycemic control presented to the ophthalmology outpatient department with a one-week history of blurred vision in the left eye (LE). The patient had previously been diagnosed with neovascular glaucoma (NVG; post-central retinal artery occlusion (CRAO)) elsewhere and was on a medication regimen including topical timolol, brimonidine, and brinzolamide, along with oral acetazolamide.

On examination, visual acuity in the left eye was severely impaired (finger counting close to the face temporally) and IOP measured 36 mmHg. Gonioscopy revealed 360 degrees of peripheral anterior synechiae (PAS) grade 2. Slit lamp examination revealed diffuse corneal stromal edema and posterior chamber intra-ocular lens (PCIOL) in situ.

The right eye's vision was 6/6, and the anterior and posterior segments were within normal limits. There was no recent history of ocular surgery or trauma. 

**Figure 1:**
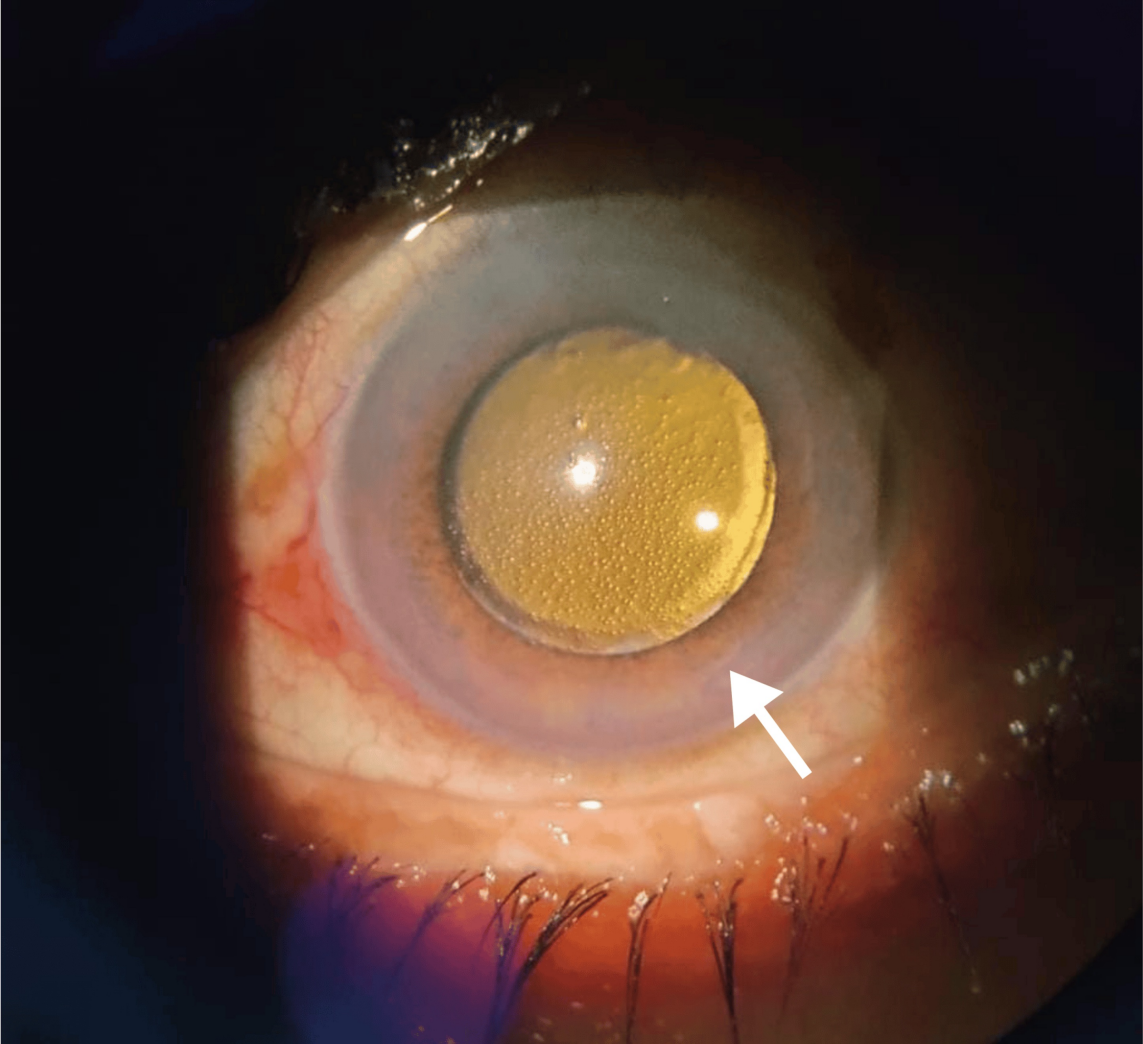
Anterior segment slit lamp photo of the left eye showing diffuse honeycomb-like cystic lesions in retro illumination.

**Figure 2:**
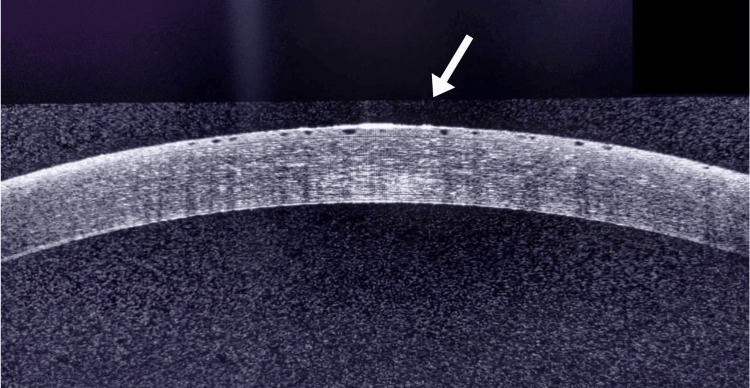
Anterior segment optical coherence tomography (AS-OCT) image of left eye showing uniform diffuse subepithelial cystic lesions

Topical netarsudil, 0.02% drops daily once was added to achieve the target IOP. One week post-therapy, the slit lamp examination revealed honeycomb-like cystic lesions over the cornea (Figure 1). Anterior segment optical coherence tomography (AS-OCT) confirmed the presence of subepithelial cystic lesions (Figure 2).

There was no history of previously used topical prostaglandin analogs. Careful slit lamp examination revealed unilateral involvement, and the fellow eye showed no endothelial guttae or pigmentation, ruling out corneal endothelial dystrophy. The onset of symptoms within a week of starting netarsudil and the absence of diurnal variation, pain, or severe congestion were in the favor of the drug-related (netarsudil) edema. Additionally, the edema presented a uniform pattern on both slit lamp and anterior segment OCT, further supporting the diagnosis. Considering these factors, a diagnosis of netarsudil drug-related corneal edema was made.

**Figure 3:**
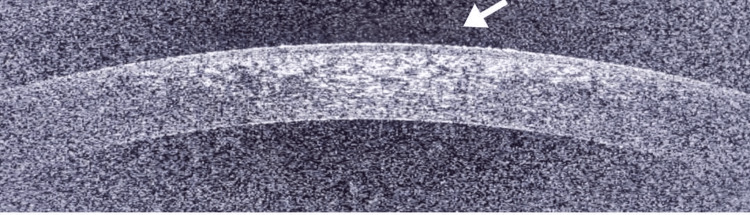
Anterior segment image of left eye in subsequent follow-up showing complete resolution of subepithelial cystic lesions

Despite this unexpected corneal complication, the patient continued the netarsudil regimen. At subsequent follow-up visits, the patient’s corneal lesions gradually resolved, with intraocular pressure remaining within acceptable limits. By the six-week follow-up, both slit-lamp examination and AS-OCT imaging showed complete resolution of the cystic lesions (Figure 3). The patient’s overall condition, including visual acuity and subjective symptoms, remained stable throughout this period.

## Discussion

ROCK inhibitors have shown strong efficacy in controlling intraocular pressure (IOP). However, with their increased use, an associated “honeycomb” pattern of reticular epitheliopathy has been documented in several new studies [5].

As in previous reports of reticular corneal epithelial edema, our patient did not have any clear predisposing risk factors, like a history of anterior uveitis, corneal transplant, Fuchs corneal dystrophy, diode laser photocoagulation, congenital glaucoma, or tube shunt . However, corneal endothelial cell count was not done before starting netarsudil eye drops.

Rho-kinase inhibitors like ripasudil and netarsudil can cause temporary morphological changes in corneal endothelial cells, disrupt epithelial tight junctions, and alter the actin cytoskeleton, potentially leading to bullae formation [6,7]. Yet, endothelial changes alone don't fully explain the reticular corneal epithelial edema observed with netarsudil. It’s proposed that netarsudil increases tight junction permeability, allowing stromal edema to extend into the epithelial layers [8].

In our patient, reticular corneal epithelial edema resolved even while netarsudil drops were continued. A similar case was reported by Tran et al. where reticular corneal edema also resolved despite the ongoing use of netarsudil eye drops [3]. They attributed this resolution to the stronger healing capacity often observed in younger individuals.

## Conclusions

As netarsudil gains traction in glaucoma management, this case highlights the importance of recognizing rare ocular side effects, such as corneal epithelial bullae. A thorough clinical examination, including slit-lamp photography and AS-OCT, is essential for monitoring. While most corneal effects appear reversible after discontinuing the medication, further research and vigilant patient monitoring remain crucial for optimal care.
